# The influence of algal biostimulator and nitrogen source on the phytochemical composition and biological properties of *Corchorus olitorius* leaves and stems

**DOI:** 10.1038/s41598-026-39774-z

**Published:** 2026-03-12

**Authors:** Wael Mahmoud Aboulthana, Amal M. El-Feky, Nagwa I. Omar, Abo El-Khair B. El-Sayed

**Affiliations:** 1 Biochemistry Department, Biotechnology Research Institute, National Research Centre, 33 El Bohouth St., Dokki, Giza, 12622 Egypt; 2 Pharmacognosy Department, Pharmaceutical and Drug Industries Research Institute, National Research Centre, 33 El Bohouth St., Dokki, Giza, 12622 Egypt; 3 Algal Biotechnology Unit, Department of Fertilization Technology, Biological, and Agricultural Research Institution, National Research Centre, 33 El Bohouth St., Dokki, Giza, 12622 Egypt

**Keywords:** *Corchorus olitorius*, Phyto-constituents, Algal biostimulator, Anti-diabetic activity, Cytotoxic activity, Biochemistry, Biotechnology, Plant sciences

## Abstract

The present study aimed to evaluate the impact of different nitrogen sources combined with an algal biostimulant on the biochemical composition and biological activities of *Corchorus olitorius* (Mulukhiyah) grown under saline irrigation. A pot experiment was conducted during the summer season of 2023 at the Algal Biotechnology Unit, National Research Centre, Cairo, Egypt. Seeds were soaked in algal slurry and cultivated in sandy soil, irrigated with *Pandorina sp.* slurry (3846.4 ppm on average), and supplemented with an in situ algal biostimulator applied as a foliar spray (1.0 mL L^−1^) in combination with nitrogen, phosphorus, and potassium (NPK) fertilization. Phytochemical analyses revealed that leaves consistently accumulated higher levels of sterols, terpenes, fatty acids, pigments, polyphenols, tannins, and flavonoids compared with stems. Among the three fertilization regimes, ammonium nitrate, in combination with the biostimulant, produced the highest concentrations of sterols (1.03% in leaves), terpenes (0.93%), fatty acids (3.05%), chlorophylls (4.16 mg/g), carotenoids (3.79 mg/g), polyphenols (252.76 mg/100 g), condensed tannins (101.10 μg/mL), and flavonoids (6.19 mg/g). HPLC profiling confirmed the presence of diverse phenolic and flavonoid compounds, including gallic acid, chlorogenic acid, caffeic acid, rutin, quercetin, and kaempferol, with leaves exhibiting a broader and higher profile than stems. In vitro biological assays demonstrated that ammonium nitrate–treated extracts exhibited superior antioxidant activity (total antioxidant capacity: 129.6 ± 2.3 mg GAE/g; DPPH scavenging IC₅₀: 41.8 ± 1.2 µg/mL), anti-Alzheimer activity (AChE inhibition: 67.4% at 200 µg/mL), anti-diabetic activity (α-amylase IC₅₀: 72.3 ± 1.5 µg/mL), and cytotoxic effects, particularly against Caco-2 cells (IC₅₀: 50.65 ± 2.84 µg/mL). Conversely, no significant enhancement was observed in anti-inflammatory and anti-arthritic properties, where all extracts showed comparable inhibition (~ 48–52%) relative to the standard drug diclofenac. Overall, the findings indicate that the combined application of ammonium nitrate fertilizer and algal biostimulant enhances the accumulation of bioactive phytochemicals in *C. olitorius*, which translates into improved pharmacological potential, particularly antioxidant and cytotoxic activities. These results support the use of optimized fertilization strategies for improving the nutraceutical and medicinal value of *C. olitorius* under saline conditions.

**Supplementary Information:**

The online version contains supplementary material available at 10.1038/s41598-026-39774-z.

## Introduction

Leafy green vegetables are an integral component of traditional diets in many low-income countries, where they serve not only as staple foods but also as sources of health-promoting bioactive compounds^1,2^. Among these, *Corchorus olitorius* L., an annual herbaceous species belonging to the family Malvaceae, is characterized by a slender stem and alternately arranged leaves with finely serrated margins^3^. Commonly referred to as Mulukhiyah, this species originated along the Mediterranean coast of Egypt and is now widely cultivated across tropical and subtropical regions^4^.

The leaves of *C. olitorius* have long been valued in traditional medicine for their nutritional and therapeutic properties^5^. In West Africa, they are frequently consumed in soups due to their richness in essential vitamins, minerals (notably calcium and magnesium), dietary fibers, and phenolic compounds^6^. Phytochemical investigations have demonstrated that *C. olitorius* contains a wide range of bioactive metabolites with antioxidant potential^7^. For instance, ionone glycosides (corchoionosides A, B, and C) inhibit histamine release, while corchorifatty acids A–F reduce nitric oxide production in mouse peritoneal macrophages^8^. Additionally, the leaves are a notable source of vitamins C, E, and A, minerals such as iron and calcium, proteins with all ten essential amino acids, mucilage, β-carotene, lutein, and several phenolic derivatives including 5-caffeoylquinic acid, quercetin, and its glycosides, highlighting their strong antioxidant potential^9,10^. Accordingly, *C. olitorius* has found wide applications in both dietary practices and medicinal product formulations^11,12^.

Beyond nutrition, *C. olitorius* leaves are traditionally consumed to enhance immunity^13^ and to promote wound healing^14^. Ethnomedicinal practices also employ them in the management of malaria and typhoid fever^15^, female infertility^6^, heart failure^16^, gastric ulcers^17^, and other conditions. These applications are supported by reports of their antioxidant^18^, antimicrobial^19^, anti-inflammatory^20^, and anticancer activities^21^. A recent review further highlighted the potential of *C. olitorius* in improving glucose metabolism and providing gastroprotective effects^22^. In addition, Shariare et al.^23^ demonstrated anti-obesity effects of *C. olitorius* leaves in high-fat diet–induced obese rats, while Do et al.^24^ reported that dietary supplementation regulated gut microbiota and reduced hepatic fat accumulation in high-fat diet–fed mice, suggesting possible benefits in metabolic disorders. In Nigeria, leaf infusions are administered to counter iron and folic acid deficiencies^15^, while in Tanzania, the plant is consumed for its laxative, diuretic, emollient, and nutritional properties, particularly in the treatment of infantile malnutrition^9^.

Importantly, the nutritional and therapeutic values of *C. olitorius* are influenced by agricultural practices, post-harvest processing, and drying techniques, which can alter the concentration and stability of bioactive metabolites^25^. Biofertilizers, consisting of beneficial microorganisms and organic materials, represent a promising approach to enhancing soil fertility, stimulating plant growth, and improving yield in an environmentally sustainable manner^26,27^. Compared to synthetic fertilizers, they are often more cost-effective and can offer superior efficiency in promoting plant health^28^.

In recent years, algal-based biostimulants have gained attention as eco-friendly supplements that not only improve crop productivity but also enrich plant phytochemical profiles. However, their synergistic use with specific nitrogen sources, and how such combinations modulate both the phytochemical composition and pharmacological potential of *C. olitorius*, remains underexplored. Therefore, this study addresses this research gap by investigating, for the first time, the combined influence of an algal biostimulant and different nitrogen fertilizers on the phytoconstituent content and biological activities of *C. olitorius* leaves and stems. This integrative approach provides novel insights into how tailored fertilization strategies can optimize the nutraceutical and medicinal value of *C. olitorius* under saline irrigation conditions.

## Materials and methods

### Plant cultivation and extraction

Seeds of *Corchorus olitorius* were obtained from the Horticulture Research Centre, Ministry of Agriculture, Egypt. No wild plants were collected for this study. All experimental procedures complied with institutional, national, and international guidelines and legislation, including the IUCN Policy Statement on Research Involving Species at Risk of Extinction and the Convention on the Trade in Endangered Species of Wild Fauna and Flora (CITES). Plants were cultivated in sandy soil at the Algal Biotechnology Unit, National Research Centre, Egypt. Plants were fertilized in situ with a formulated NPK fertilizer (10:05:05) and treated with an algal biostimulator to enhance the accumulation of phytoconstituents. Three nitrogen sources were applied in separate treatments: (1) urea, (2) ammonium sulfate, and (3) ammonium nitrate.

The green microalga *Chlorella vulgaris* was cultured in a 75 m^3^ open pond following El-Sayed et al.^29^, under a salinity level of 3846.4 ppm, and subsequently used for irrigation. A portion of the *Chlorella* culture was harvested and processed to prepare the algal biostimulant according to Anter and El-Sayed^30^. In addition, an in situ prepared NPK fertilizer (5:5:20) and an algal biostimulator derived from the green alga *Pandorina sp.* were applied as foliar sprays at a concentration of 1.0 mL L⁻^1^.

At the end of the cultivation season (January 2024), fresh leaves and stems of *C. olitorius* were harvested. Taxonomical identification was confirmed by Prof. Gamal Farag (Horticulture Research Centre, Ministry of Agriculture, Egypt), and a voucher specimen was deposited in the National Research Centre Herbarium (Voucher no. 214). The plant material was washed thoroughly, air-dried at 45 °C to a constant weight, ground separately into fine powders (leaves and stems), labeled, and stored in airtight glass containers at − 20 °C until use.

For each agricultural treatment, 200 g of powdered leaves and stems were subjected separately to extraction following the method of Meneses et al.^31^, using chloroform–methanol (2:1, v/v) to obtain a wide range of phytoconstituents. The extracts were concentrated under reduced pressure using a rotary evaporator, and the residues were stored in tightly sealed dark glass vials at 4 °C until further analysis.

### Phyto-chemical profile

#### Quantitative determination of total sterols, terpenes and fatty acids

The total sterol and terpene contents of *C. olitorius* leaf and stem extracts were quantified spectrophotometrically using the Liebermann–Burchard reaction^32^. The assay measured the color intensity produced after reaction with the reagent, and concentrations were determined by comparison with standard calibration curves of stigmasterol and α-amyrin. The color reaction was allowed to develop in the dark for 30 min, after which the absorbance was measured at 625 nm against a reagent blank^33^.

For fatty acid determination, the extracts were refluxed separately with 0.5 N alcoholic KOH for 6 h^34^. After cooling, the mixtures were diluted with an equal volume of distilled water and extracted thoroughly with diethyl ether. The remaining aqueous alkaline layer was acidified with HCl to liberate free fatty acids, which were subsequently extracted several times with ether. The combined ether extracts were washed repeatedly with distilled water until neutral, dried over anhydrous sodium sulfate, and evaporated to dryness. The residues were weighed, and the yield was expressed as the percentage of total free fatty acids^35^.

#### Pigment content

Chlorophyll *a* and chlorophyll *b* contents were determined spectrophotometrically at 664 nm and 651 nm, respectively, while total carotenoids were measured at 470 nm. Total chlorophylls, total carotenoids, the chlorophyll *a/b* ratio, and the pigment index (carotenoids/chlorophyll *a*) were calculated using the equations described by Elbatanony et al.^36^.

#### Quantitative determination of total polyphenols, tannins and flavonoids

The concentrations of polyphenols, condensed tannins, and flavonoids in *C. olitorius* leaf and stem extracts were determined following standard protocols. Total polyphenols were quantified using the Folin–Ciocalteu method^37^ and expressed as mg gallic acid equivalents (GAE) per 100 g dry weight^38^. Condensed tannins were estimated according to Broadhurst and Jones^39^ and expressed as μg/mL. The total flavonoid content was determined according to the method of Madaan et al.^40^, which involves the formation of a flavonoid–aluminum chloride complex and measurement of the resulting color intensity. The concentration was quantified against a standard calibration curve prepared with quercetin and expressed as milligrams of quercetin equivalents (QE) per 100 g of dry weight^41^.

#### HPLC identification of phenolics and flavonoids

Phenolic and flavonoid constituents were analyzed by HPLC (Agilent 1260, NRC Central Laboratories, Cairo, Egypt) using an Eclipse C18 column (4.6 × 250 mm, 5 μm). The mobile phase comprised water (A) and acetonitrile with 0.05% trifluoroacetic acid (B), applied in a linear gradient: 0–5 min (82–80% A), 5–12 min (60% A), and re-equilibration to 82% A until 20 min. The flow rate was 0.9 mL/min, injection volume 5 μL, and column temperature 40 °C. Compounds were detected at 280 nm and identified by comparing retention times with authentic standards^42^.

### In vitro biological activities

For in vitro biological assays, the experiments were conducted following a completely randomized design (CRD). Extracts were coded and analyzed in a randomized order to reduce experimental bias. All assays were performed in triplicate (technical replicates) and repeated in three independent biological experiments, and the results are presented as mean ± SE.

#### Antioxidant activity

The total antioxidant capacity (TAC) was determined using the method of Prieto et al.^43^. Results were expressed as milligrams of gallic acid equivalent per gram of extract, with ascorbic acid applied as the standard at equivalent concentrations^44^. The iron reducing power (IRP) was evaluated according to Oyaizu^45^ and expressed in µg/mL. An increase in absorbance at 700 nm was interpreted as higher reducing power, with ascorbic acid serving as the reference standard.

#### Scavenging activity

The DPPH radical scavenging activity was measured according to Rahman et al.^46^, using ascorbic acid as a positive control. The percentage of DPPH inhibition was calculated, and the half-maximal inhibitory concentration (IC₅₀) was obtained from the dose–response curve plotted between extract concentration and inhibition percentage. The ABTS radical scavenging assay was performed following the procedure of Arnao et al.^47^. The scavenging activities of the extracts were compared with that of ascorbic acid. The percentage of ABTS radical inhibition was calculated, and IC₅₀ values were derived from inhibition curves prepared at different sample concentrations.

#### Anti-Alzheimer activity

The inhibitory activity against acetylcholinesterase (AChE; electric eel, Type VI-S, Sigma-Aldrich, USA) was determined using Ellman’s method^48^. The reaction mixture (200 µL) contained 0.1 M phosphate buffer (pH 8.0), 0.5 mM 5,5′-dithiobis-(2-nitrobenzoic acid) (DTNB), and 0.5 mM acetylthiocholine iodide as substrate. Various concentrations of extracts (10–200 µg/mL) were pre-incubated with the enzyme at 25 °C for 15 min before substrate addition. Absorbance was measured at 412 nm using a microplate reader. Donepezil served as the positive control. IC₅₀ values were calculated from inhibition curves.

#### Anti-diabetic activity

The inhibition of α-amylase and α-glucosidase was evaluated as described by Wickramaratne et al.^49^ and Pistia-Brueggeman and Hollingsworth^50^, with modifications. **α-Amylase inhibition:** The enzyme (porcine pancreatic α-amylase, Sigma-Aldrich) was prepared in 0.1 M phosphate buffer (pH 6.8). Extracts (10–200 µg/mL) were incubated with the enzyme (1 U/mL) at 37 °C for 10 min before adding 1% soluble starch solution as the substrate. After 30 min at 37 °C, the reaction was stopped with 3,5-dinitrosalicylic acid reagent, boiled for 5 min, cooled, and absorbance was measured at 540 nm. **α-Glucosidase inhibition:** Yeast α-glucosidase (Sigma-Aldrich) was dissolved in 0.1 M phosphate buffer (pH 6.8). Extracts (10–200 µg/mL) were pre-incubated with the enzyme (1 U/mL) at 37 °C for 10 min before adding 5 mM p-nitrophenyl-α-D-glucopyranoside (pNPG) as substrate. After 20 min, absorbance was measured at 405 nm. Acarbose (10–100 µM) was used as the positive control in both assays. IC₅₀ values were calculated from inhibition curves.

#### Anti-arthritic activity

Two in vitro models were employed: protein denaturation^51^ and proteinase inhibition^52^. **Protein denaturation:** The reaction mixture contained 0.45 mL of bovine serum albumin (5% aqueous solution) and 0.05 mL of extract solution (10–200 µg/mL) in phosphate buffer (pH 6.3). Samples were incubated at 37 °C for 20 min and then heated at 70 °C for 5 min. After cooling, turbidity was measured at 660 nm. **Proteinase inhibition:** Trypsin (0.06 mg/mL, Sigma-Aldrich) was prepared in Tris–HCl buffer (20 mM, pH 7.4) with 0.02 M CaCl₂. Extracts (10–200 µg/mL) were pre-incubated with the enzyme at 37 °C for 5 min, followed by addition of casein (0.8% w/v) as the substrate. After 20 min at 37 °C, the reaction was terminated with 70% perchloric acid, centrifuged, and absorbance was measured at 280 nm. Diclofenac sodium was used as the reference drug. IC₅₀ values were derived from inhibition curves.

#### Anti-inflammatory activity

Cyclooxygenase (COX-1 and COX-2; ovine/human, Cayman Chemical) and 5-lipoxygenase (5-LOX; human recombinant, Cayman Chemical) inhibition assays were carried out using commercial colorimetric kits. Extracts (10–200 µg/mL) were incubated with the respective enzymes according to the manufacturer’s instructions. COX activity was monitored by measuring prostaglandin production at 590 nm^53^. While 5-LOX activity was monitored by following the formation of 5-hydroperoxyeicosatetraenoic acid (5-HPETE) at 490 nm. Indomethacin (COX) and zileuton (5-LOX) served as positive controls. IC₅₀ values were calculated from inhibition curves^54^.

#### Cytotoxic activity

Cytotoxic effects were evaluated using the MTT assay^55^. Human hepatocellular carcinoma (HepG2), colon carcinoma (Caco-2), and lung carcinoma (A549) cells were obtained from Nawah Scientific Inc. (Cairo, Egypt). In addition, normal human dermal fibroblasts (HDF) were used to assess the selectivity and potential safety of the extracts.Cells were seeded at a density of 1 × 10^4^ cells/well in 96-well plates and allowed to attach overnight. Extracts (10–200 µg/mL) were added and incubated for 48 h at 37 °C in 5% CO₂. After treatment, 20 µL of MTT solution (5 mg/mL) was added per well and incubated for 4 h. Formazan crystals were dissolved with 100 µL DMSO, and absorbance was measured at 570 nm. Doxorubicin (1 µM) served as the positive control^56^. Cell viability was expressed as percentage growth inhibition, and IC₅₀ values were calculated.

#### The enzymatic activity

The effect of extracts on apoptosis-related enzymes was investigated by measuring caspase-3 activity and Bcl-2 expression in HepG2, Caco-2, and A549 cells^57,58^. **Caspase-3 activity:** Measured using a colorimetric assay kit (Abcam, UK) based on the cleavage of the Ac-DEVD-pNA substrate. Cells were treated with extracts (10–100 µg/mL) for 24 h, lysed, and incubated with the substrate at 37 °C for 2 h. Absorbance was read at 405 nm. **Bcl-2 expression:** Determined using an ELISA kit (Thermo Fisher Scientific, USA) according to the manufacturer’s instructions. Treated cells were lysed, and protein levels were quantified at 450 nm. Staurosporine (1 µM) served as the positive control. Results were expressed as fold-change compared to untreated cells.

### Statistical analysis

All experiments were performed in triplicate, and data were expressed as mean ± standard error (SE). Statistical analyses were conducted using SPSS software version 25 (IBM Corp., Armonk, NY, USA). One-way analysis of variance (ANOVA) was applied to assess significant differences among treatments, followed by Tukey’s post hoc test for multiple comparisons. IC₅₀ values were determined by nonlinear regression analysis using GraphPad Prism version 9.5 (GraphPad Software, San Diego, CA, USA). Differences were considered statistically significant at *p* < 0.05.

## Results

### Phyto-chemical profile

#### Total sterols, terpenes and fatty acids

The distribution of sterols, terpenes, and fatty acids in the six extracts obtained from *C. olitorius* leaves and stems under different agricultural treatments is presented in Table 1. In the first treatment (urea), the sterol content was 0.97% in leaves and 0.57% in stems. The second treatment (ammonium sulfate) resulted in lower sterol values of 0.86% and 0.49% for leaves and stems, respectively. By contrast, the third treatment (ammonium nitrate) showed the highest sterol content, reaching 1.03% in leaves and 0.68% in stems. A similar trend was observed for terpenes. In treatment 1, leaves and stems contained 0.81% and 0.51%, respectively, while treatment 2 recorded slightly lower values (0.74% and 0.46%). Treatment 3 displayed the highest terpene content (0.93% in leaves and 0.62% in stems). Regarding fatty acids, the third treatment again recorded the highest values (3.05% in leaves and 1.87% in stems), compared to treatment 1 (2.39% and 1.46%) and treatment 2 (2.47% and 1.38%). Overall, the leaves accumulated higher concentrations of sterols, terpenes, and fatty acids than stems across all treatments.

**Table 1:** Total sterols, terpenes, and fatty acids (%) in *C. olitorius* leaves and stems under the three agricultural treatments.

Phyto-constituents	%					
	Urea		Ammonium sulfate		Ammonium nitrate	
	Leaves	Stems	Leaves	Stems	Leaves	Stems
Sterols	0.97 ± 0.21	0.57 ± 0.14	0.86 ± 0.05	0.49 ± 0.03	1.03 ± 0.10	0.68 ± 0.31
Terpenes	0.81 ± 0.16	0.51 ± 0.10	0.74 ± 0.27	0.46 ± 0.14	0.93 ± 0.06	0.62 ± 0.15
Fatty acids	2.39 ± 0.07	1.46 ± 0.05	2.47 ± 0.12	1.38 ± 0.07	3.05 ± 0.11	1.87 ± 0.09

Data presented as mean ± SE, n = 3.

#### Pigment content

The pigment content of *C. olitorius* leaves and stems under three fertilization treatments is presented in Table 2. Among the treatments, ammonium nitrate (Treatment 3) resulted in the highest levels of chlorophylls and carotenoids, while ammonium sulfate (Treatment 2) showed the lowest. In leaves, chlorophyll *a* and *b* concentrations in Treatment 3 were 2.87 and 1.14 mg/g, respectively, compared with 2.69 and 1.09 mg/g in Treatment 1 (urea) and 2.38 and 1.06 mg/g in Treatment 2. In stems, chlorophyll *a* levels were 1.92, 1.73, and 1.70 mg/g for Treatments 3, 1, and 2, respectively, while chlorophyll *b* levels were 0.75, 0.69, and 0.67 mg/g. Total chlorophyll content was also highest under Treatment 3, reaching 4.16 mg/g in leaves and 2.73 mg/g in stems, compared with 3.89 and 2.57 mg/g (Treatment 1) and 3.51 and 2.43 mg/g (Treatment 2). Consequently, plants under Treatment 3 developed a visibly darker green color. Carotenoid concentrations followed a similar trend. Treatment 3 yielded the highest total carotenoid content, with 3.79 mg/g in leaves and 2.65 mg/g in stems, compared to 3.26 and 2.43 mg/g in Treatment 1 and 3.02 and 2.16 mg/g in Treatment 2.

**Table 2:** Pigment contents in *C. olitorius* leaves and stems under the three agricultural treatments.

Pigment content	Concentration (mg/g)					
	Urea		Ammonium sulfate		Ammonium nitrate	
	Leaves	Stems	Leaves	Stems	Leaves	Stems
Chlorophyll a	2.69 ± 0.03	1.73 ± 0.02	2.38 ± 0.16	1.70 ± 0.24	2.87 ± 0.13	1.92 ± 0.09
Chlorophyll b	1.09 ± 0.12	0.69 ± 0.14	1.06 ± 0.10	0.67 ± 0.08	1.14 ± 0.05	0.75 ± 0.11
Chlorophylls a/b ratio	2.47 ± 0.10	2.51 ± 0.09	2.24 ± 0.08	2.54 ± 0.13	2.52 ± 0.10	2.56 ± 0.07
Total chlorophylls	3.89 ± 0.04	2.57 ± 0.06	3.51 ± 0.12	2.43 ± 0.25	4.16 ± 0.34	2.73 ± 0.16
Total carotenoids	3.26 ± 0.15	2.43 ± 0.42	3.02 ± 0.22	2.16 ± 0.17	3.79 ± 0.03	2.65 ± 0.41
Pigment index	1.21 ± 0.01	1.40 ± 0.03	1.27 ± 0.10	1.27 ± 0.11	1.32 ± 0.05	1.38 ± 0.15

Data presented as mean ± SE, n = 3.

#### Total polyphenols, tannins and flavonoids

Significant variations in the concentrations of total polyphenols, tannins, and flavonoids were recorded in the leaves and stems of *C. olitorius* across the three agricultural treatments (Table 3). In the **urea treatment**, polyphenol concentrations reached 202.61 mg gallic acid/100 g in the leaves and 150.08 mg/100 g in the stems. The **ammonium sulfate treatment** yielded comparatively lower polyphenol levels (169.99 mg/100 g in the leaves and 125.92 mg/100 g in the stems). By contrast, the **ammonium nitrate treatment** resulted in the highest polyphenol contents, with 252.76 mg/100 g in leaves and 187.23 mg/100 g in stems.

**Table 3:** Major phyto-constituents in *C. olitorius* leaves and stems under the three agricultural treatments.

Agricultural treatment		Total polyphenols (mg gallic acid/100 g)	Total condensed tannins (μg/mL)	Total flavonoids (mg quercetin/g)
Urea	Leaves	202.61 ± 0.23	81.05 ± 0.09	5.68 ± 0.06
	Stems	150.08 ± 0.17	60.03 ± 0.07	2.46 ± 0.13
Ammonium sulfate	Leaves	169.99 ± 0.14	68.00 ± 0.06	4.96 ± 0.15
	Stems	125.92 ± 0.10	50.37 ± 0.04	1.85 ± 0.08
Ammonium nitrate	Leaves	252.76 ± 0.09	101.10 ± 0.04	6.19 ± 0.11
	Stems	187.23 ± 0.07	74.89 ± 0.03	2.97 ± 0.20

Data presented as mean ± SE, n = 3.

A similar pattern was observed in condensed tannins and flavonoids. For tannins, values were 81.05 μg/mL (leaves) and 60.03 μg/mL (stems) under urea, 68.00 μg/mL (leaves) and 50.37 μg/mL (stems) under ammonium sulfate, and peaked at 101.10 μg/mL (leaves) and 74.89 μg/mL (stems) under ammonium nitrate.

Flavonoid content followed the same trend, with 5.68 mg quercetin/g (leaves) and 2.46 mg/g (stems) under urea, 4.96 mg/g (leaves) and 1.85 mg/g (stems) under ammonium sulfate, and the highest levels of 6.19 mg/g (leaves) and 2.97 mg/g (stems) under ammonium nitrate.

#### HPLC identification of phenolics and flavonoids

**Fig. 1:**
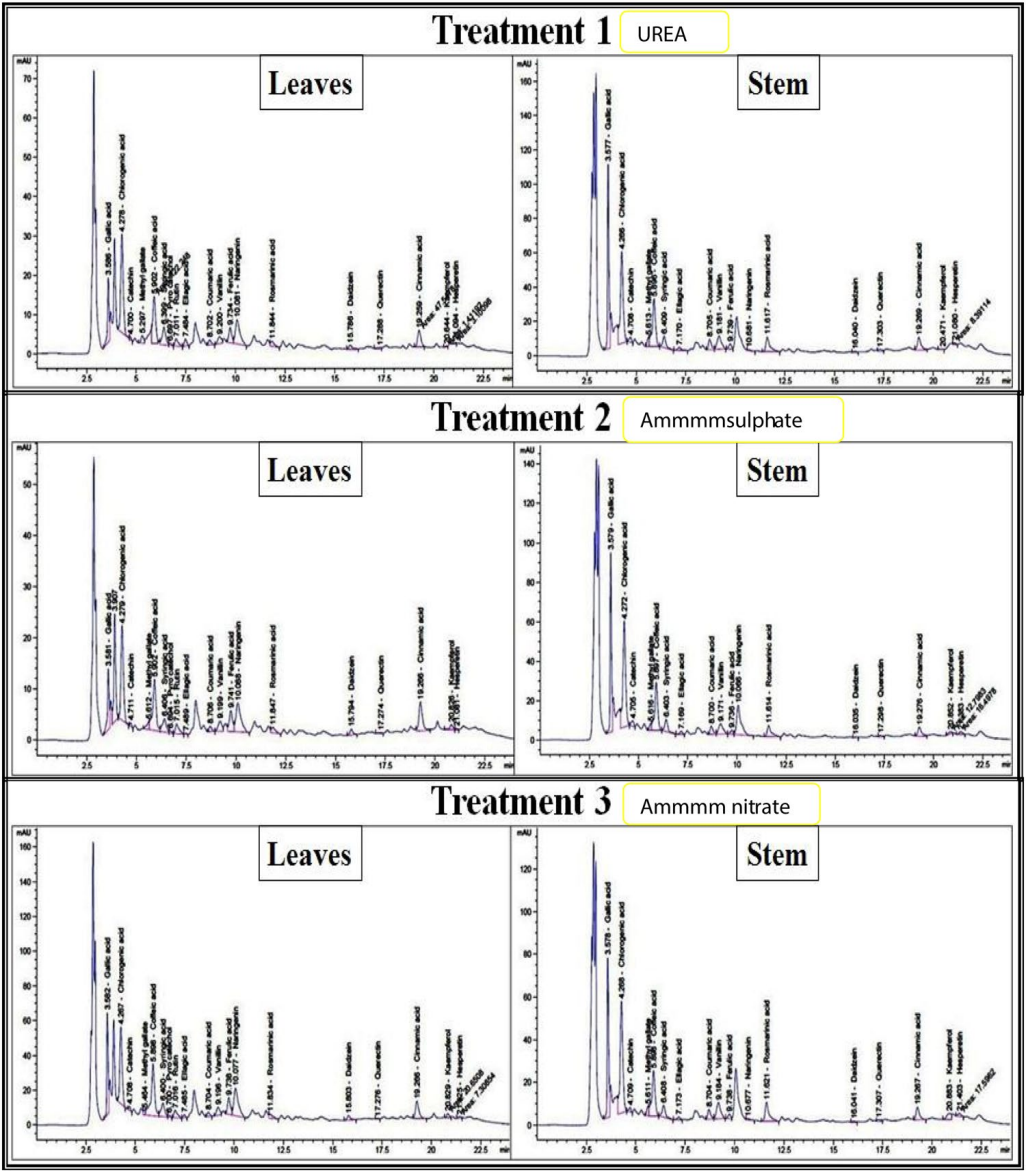
HPLC chromatogram of each extract from *C. olitorius* leaves and stems in the three agricultural treatments.

HPLC analysis revealed that *C. olitorius* is rich in diverse phenolic and flavonoid compounds, with higher concentrations in leaves than in stems across all treatments (Table 4). Treatment with ammonium nitrate consistently produced the highest levels of phenolics and flavonoids, followed by urea, while ammonium sulfate resulted in the lowest levels. Major identified compounds included gallic acid, chlorogenic acid, catechin, caffeic acid, syringic acid, rutin, ferulic acid, quercetin, kaempferol, and naringenin. Some compounds, such as pyrocatechol and rutin, were detected exclusively in leaves. Representative HPLC chromatograms are shown in Fig. 1.

**Table 4:** HPLC analysis for phenolics and flavonoids in the extracts of *C. olitorius* leaves and stems under the three agricultural treatments.

Compound	Rt (min.), Concentration(µg/g)					
	Urea		Ammonium sulfate		Ammonium nitrate	
	Leaves	Stems	Leaves	Stems	Leaves	Stems
Gallic acid	3.586 (408.68)	3.577 (1978.79)	3.581 (317.22)	3.579 (1645.21)	3.582 (1227.85)	3.578 (1981.99)
Chlorogenic acid	4.278 (1447.90)	4.266 (3381.51)	4.279 (1058.43)	4.272 (3307.37)	4.267 (2443.84)	4.268 (3526.26)
Catechin	4.700 (44.46)	4.708 (290.60)	4.711 (43.27)	4.705 (173.66)	4.708 (143.35)	4.709 (284.75)
Methyl gallate	5.297 (42.79)	5.613 (52.60)	5.612 (34.10)	5.616 (74.83)	5.464 (42.69)	5.611 (94.75)
Caffeic acid	5.902 (473.01)	5.896 (1019.00)	5.902 (463.78)	5.897 (924.04)	5.898 (1458.53)	5.898 (1305.78)
Syringic acid	6.399 (207.01)	6.409 (244.76)	6.406 (132.26)	6.403 (233.86)	6.400 (373.43)	6.408 (274.55)
Pyro catechol	6.697 (36.11)	0.00	6.694 (28.30)	0.00	6.700 (77.07)	0.00
Rutin	7.011 (154.49)	0.00	7.015 (174.48)	0.00	7.016 (389.35)	0.00
Ellagic acid	7.484 (72.37)	7.170 (69.02)	7.489 (5.22)	7.169 (79.92)	7.485 (67.48)	7.173 (83.97)
Coumaric acid	8.702 (22.22)	8.705 (79.57)	8.706 (16.29)	8.700 (85.28)	8.704 (38.73)	8.704 (92.81)
Vanillin	9.200 (42.61)	9.181 (152.81)	9.199 (47.77)	9.171 (202.44)	9.196 (97.49)	9.184 (216.02)
Ferulic acid	9.734 (110.95)	9.739 (55.17)	9.741 (139.27)	9.736 (59.50)	9.738 (278.81)	9.738 (60.34)
Naringenin	10.081 (453.24)	10.681 (1009.99)	10.088 (455.28)	10.066 (237.95)	10.077 (1139.68)	8.27 (1210.67)
Rosmarinic acid	11.844 (86.41)	11.617 (445.88)	11.847 (80.60)	11.614 (674.01)	11.834 (161.34)	11.627 (725.91)
Daidzein	15.786 (33.20)	16.040 (9.11)	15.794 (31.75)	16.035 (11.98)	15.803 (91.11)	16.041 (14.58)
Querectin	17.288 (18.92)	17.303 (69.73)	17.274 (10.55)	17.298 (83.38)	17.276 (15.74)	17.307 (83.75)
Cinnamic acid	19.259 (42.57)	19.269 (55.51)	19.266 (63.71)	19.276 (63.00)	19.266 (106.40)	19.267 (96.06)
Kaempferol	20.644 (4.45)	20.471 (40.37)	20.826 (33.86)	20.852 (136.63)	20.829 (65.13)	20.883 (141.35)
Hesperetin	21.094 (7.62)	21.080 (40.56)	21.081 (2.54)	21.383 (43.26)	21.425 (17.97)	21.403 (48.39)

### In vitro biological activities

The extracts were also assessed for their biological activities, including antioxidant, radical scavenging, anti-Alzheimer’s, anti-diabetic, anti-arthritic, and anti-inflammatory activities, and were compared to the standard drug used in each assay^59^.

#### Antioxidant and scavenging activities

The antioxidant capacity of *C. olitorius* extracts was assessed through total antioxidant capacity (TAC) and iron reducing power (IRP) assays. As shown in Table 5, both leaves and stems demonstrated considerable antioxidant activity under the three agricultural treatments. The ammonium nitrate treatment (third treatment) resulted in the highest TAC and IRP values, with the leaves (219.79 ± 0.08 and 209.32 ± 0.07 µg/mL, respectively) outperforming the stems (162.81 ± 0.06 and 155.05 ± 0.05 µg/mL, respectively). In contrast, the extracts obtained from ammonium sulfate treatment displayed the lowest TAC and IRP activities.

**Table 5:** The in vitro antioxidant activity of *C. olitorius* leaves and stems under the three agricultural treatments.

Agricultural treatment		TAC (mg gallic acid/gm)	IRP (µg/mL)
Urea	Leaves	176.19 ± 0.20^b^	167.80 ± 0.19^b^
	Stems	130.51 ± 0.15^c^	124.29 ± 0.14^c^
Ammonium sulfate	Leaves	147.82 ± 0.12^c^	140.78 ± 0.12^c^
	Stems	109.49 ± 0.09^d^	104.28 ± 0.09^d^
Ammonium nitrate	Leaves	219.79 ± 0.08^a^	209.32 ± 0.07^a^
	Stems	162.81 ± 0.06^b^	155.05 ± 0.05^b^

Data presented as mean ± SE, n = 3, Different superscript letters within each column indicate significant differences among treatments at *p* < 0.05.

Similarly, radical scavenging activity was evaluated using DPPH and ABTS assays (Table 6). The extracts from leaves and stems grown under ammonium nitrate treatment showed the strongest scavenging activities, with inhibition percentages of 60.37 ± 0.31% and 70.63 ± 0.36% for leaves, and 47.54 ± 0.24% and 55.62 ± 0.28% for stems, respectively. Corresponding IC_50_ values were significantly lower in these extracts (5.19 ± 0.02 and 3.84 ± 0.02 µg/mL for leaves; 7.28 ± 0.02 and 5.40 ± 0.01 µg/mL for stems), indicating greater potency. Ascorbic acid, the reference standard, showed the highest scavenging capacity with inhibition values of 65.41 ± 0.02% and 76.53 ± 0.03%, and the lowest IC_50_ values (4.27 ± 0.03 and 3.16 ± 0.02 µg/mL).

**Table 6:** The in vitro scavenging activity of *C. olitorius* leaves and stems under the three agricultural treatments.

Agricultural treatment		DPPH		ABTS	
		IC_50_ (µg/mL)	Inhibition (%)	IC_50_ (µg/mL)	Inhibition (%)
Urea	Leaves	10.26 ± 0.01^b^	48.30 ± 0.25^c^	7.60 ± 0.01^b^	56.51 ± 0.29^c^
	Stems	11.27 ± 0.02^c^	38.64 ± 0.20^d^	8.35 ± 0.01^c^	45.20 ± 0.23^d^
Ammonium sulfate	Leaves	14.59 ± 0.01^d^	38.64 ± 0.20^d^	10.81 ± 0.01^d^	45.20 ± 0.23^d^
	Stems	18.24 ± 0.01^e^	30.91 ± 0.16^e^	13.51 ± 0.01^e^	36.16 ± 0.18^e^
Ammonium nitrate	Leaves	5.19 ± 0.02^a^	60.37 ± 0.31^b^	3.84 ± 0.02^a^	70.63 ± 0.36^b^
	Stems	7.28 ± 0.02^b^	47.54 ± 0.24^c^	5.40 ± 0.01^b^	55.62 ± 0.28^c^
Ascorbic acid		4.27 ± 0.03^a^	65.41 ± 0.02^a^	3.16 ± 0.02^a^	76.53 ± 0.03^a^

Data presented as mean ± SE, n = 3, Different superscript letters within each column indicate significant differences among treatments at *p* < 0.05.

#### Anti-Alzheimer’s activity

The inhibitory activity of *C. olitorius* extracts against acetylcholinesterase (AChE) was evaluated, and the results are presented in Table 7. Among the three agricultural treatments, the ammonium nitrate treatment (third treatment) demonstrated the strongest inhibitory effect. The leaf extract exhibited the highest inhibition (59.78 ± 0.04%) with an IC_50_ value of 4.54 ± 0.02 µg/mL, while the stem extract showed moderate inhibition (27.19 ± 0.18%) with an IC_50_ value of 6.36 ± 0.02 µg/mL.

**Table 7:** The in vitro anti-Alzheimer’s activity of *C. olitorius* leaves and stems under the three agricultural treatments.

Agricultural treatment		IC_50_ (µg/mL)	Inhibition (%)
Urea	Leaves	8.96 ± 0.01^c^	52.28 ± 0.04^c^
	Stems	9.85 ± 0.02^c^	20.53 ± 0.15^e^
Ammonium sulfate	Leaves	12.75 ± 0.01^d^	44.78 ± 0.04^d^
	Stems	15.94 ± 0.01^e^	14.74 ± 0.12^f^
Ammonium nitrate	Leaves	4.54 ± 0.02^b^	59.78 ± 0.04^b^
	Stems	6.36 ± 0.02^b^	27.19 ± 0.18^d^
Donepezil		3.73 ± 0.03^a^	74.14 ± 0.02^a^

Data presented as mean ± SE, n = 3, Different superscript letters within each column indicate significant differences among treatments at *p* < 0.05.

In comparison, extracts obtained from plants grown with urea and ammonium sulfate displayed weaker inhibitory effects, with IC_50_ values ranging from 8.96 to 15.94 µg/mL. The reference drug donepezil showed the highest anti-Alzheimer’s activity, achieving 74.14 ± 0.02% inhibition and the lowest IC_50_ value (3.73 ± 0.03 µg/mL), thus outperforming all tested extracts.

#### Anti-diabetic activity

The inhibitory effects of *C. olitorius* extracts against α-amylase and α-glucosidase were evaluated, and the results are presented in Table 8. Among the three agricultural treatments, the ammonium nitrate treatment (third treatment) demonstrated the strongest enzyme inhibition. The leaf extract exhibited inhibition of 56.51 ± 0.29% (α-amylase) and 46.01 ± 0.29% (α-glucosidase), with IC_50_ values of 2.85 ± 0.01 and 2.11 ± 0.01 µg/mL, respectively. The stem extract showed moderate inhibition of 44.49 ± 0.23% and 33.99 ± 0.23%, with IC_50_ values of 4.00 ± 0.01 and 2.96 ± 0.01 µg/mL, respectively.

**Table 8:** The in vitro anti-diabetic activity of *C. olitorius* leaves and stems under the three agricultural treatments.

Agricultural treatment		α-Amylase		α-Glucosidase	
		IC_50_ (µg/mL)	Inhibition (%)	IC_50_ (µg/mL)	Inhibition (%)
Urea	Leaves	5.63 ± 0.00^c^	45.20 ± 0.23^c^	4.17 ± 0.00^c^	34.70 ± 0.23^c^
	Stems	6.19 ± 0.01^c^	36.16 ± 0.18^d^	4.58 ± 0.01^c^	25.66 ± 0.18^d^
Ammonium sulfate	Leaves	8.01 ± 0.01^d^	36.16 ± 0.18^d^	5.93 ± 0.00^d^	25.66 ± 0.18^d^
	Stems	10.01 ± 0.01^e^	28.93 ± 0.15^e^	7.41 ± 0.00^e^	18.43 ± 0.15^e^
Ammonium nitrate	Leaves	2.85 ± 0.01^b^	56.51 ± 0.29^b^	2.11 ± 0.01^b^	46.01 ± 0.29^b^
	Stems	4.00 ± 0.01^b^	44.49 ± 0.23^c^	2.96 ± 0.01^b^	33.99 ± 0.23^c^
Acarbose		2.34 ± 0.02^a^	68.28 ± 0.04^a^	1.74 ± 0.01^a^	57.78 ± 0.04^a^

Data presented as mean ± SE, n = 3, Different superscript letters within each column indicate significant differences among treatments at *p* < 0.05.

In contrast, extracts obtained from urea and ammonium sulfate treatments displayed weaker inhibitory effects, with IC_50_ values ranging from 5.63 to 10.01 µg/mL for α-amylase and 4.17 to 7.41 µg/mL for α-glucosidase. The standard drug acarbose showed the strongest inhibitory activity, with 68.28 ± 0.04% and 57.78 ± 0.04% inhibition, and the lowest IC50 values (2.34 ± 0.02 and 1.74 ± 0.01 µg/mL).

#### Anti-arthritic activity

The anti-arthritic activity of *C. olitorius* extracts was assessed through protein denaturation and proteinase inhibitory assays (Table 9). Across all three agricultural treatments, both leaf and stem extracts exhibited comparable levels of inhibition, with no significant variations in their percentages or IC_50_ values. This indicates that the extracts showed weak or negligible anti-arthritic activity. In contrast, the reference drug diclofenac sodium displayed markedly stronger activity, with inhibition values of 48.78 ± 0.02% (protein denaturation) and 46.58 ± 0.02% (proteinase inhibition), along with the lowest IC_50_ value (6.48 ± 0.03 µg/mL), clearly outperforming all *C. olitorius* extracts.

**Table 9:** The in vitro anti-arthritic activity of *C. olitorius* leaves and stems under the three agricultural treatments.

Agricultural treatment		Proteinase denaturation	Proteinase activity	
		Inhibition (%)	IC_50_ (µg/mL)	Inhibition (%)
Urea	Leaves	25.29 ± 0.02^c^	17.94 ± 0.02^c^	22.79 ± 0.02^c^
	Stems	25.64 ± 0.02^c^	18.29 ± 0.02^c^	23.14 ± 0.02^c^
Ammonium sulfate	Leaves	25.61 ± 0.03^c^	18.26 ± 0.03^c^	23.11 ± 0.03^c^
	Stems	25.99 ± 0.02^c^	18.64 ± 0.02^c^	23.49 ± 0.02^c^
Ammonium nitrate	Leaves	25.96 ± 0.03^c^	18.61 ± 0.03^c^	23.46 ± 0.03^c^
	Stems	26.34 ± 0.02^c^	18.99 ± 0.02^c^	23.84 ± 0.02^c^
Diclofenac sodium		48.78 ± 0.02^a^	6.48 ± 0.03^b^	46.58 ± 0.02^a^

Data presented as mean ± SE, n = 3, Different superscript letters within each column indicate significant differences among treatments at *p* < 0.05.

#### Anti-inflammatory activity

The inhibitory effects of *C. olitorius* extracts on cyclooxygenase (COX-1, COX-2) and lipoxygenase (5-LOX) enzymes were evaluated after the three agricultural treatments (Table 10). The leaf and stem extracts exhibited similar levels of inhibition across treatments, with consistent percentages and IC_50_ values, indicating weak or negligible anti-inflammatory activity. For COX-1 inhibition, leaf extracts showed inhibition rates around 35–36% (IC_50_ ≈ 14 µg/mL), while stem extracts displayed weaker inhibition at ~ 23–24% (IC_50_ ≈ 21 µg/mL). A similar pattern was observed for COX-2, where leaves achieved ~ 40–41% inhibition (IC_50_ ≈ 10 µg/mL), and stems ~ 28–29% inhibition (IC_50_ ≈ 15 µg/mL). For 5-LOX inhibition, leaf extracts inhibited ~ 29–30% of activity (IC_50_ ≈ 16 µg/mL), while stem extracts inhibited ~ 17–18% (IC_50_ ≈ 27 µg/mL). In contrast, the reference drug indomethacin showed potent inhibitory activity against COX-1 and COX-2 (76.77 ± 0.47% and 81.52 ± 0.47%, respectively), with the lowest IC_50_ values (6.73 ± 0.05 and 5.31 ± 0.07 µg/mL). Zileuton, used as the reference for 5-LOX, exhibited the highest inhibition of this enzyme (70.77 ± 0.47%) with the lowest IC_50_ value (6.79 ± 0.13 µg/mL).

**Table 10:** The in vitro anti-inflammatory activity of *C. olitorius* leaves and stems under the three agricultural treatments.

Agricultural treatment		COX-1		COX-2		5-LOX	
		IC_50_ (µg/mL)	Inhibition (%)	IC_50_ (µg/mL)	Inhibition (%)	IC_50_ (µg/mL)	Inhibition (%)
Urea	Leaves	14.46 ± 0.22^c^	35.77 ± 0.36^c^	10.68 ± 0.08^c^	40.52 ± 0.36^c^	16.17 ± 0.57^c^	29.77 ± 0.36^c^
	Stems	21.68 ± 0.33^c^	23.84 ± 0.24^c^	15.13 ± 0.12^c^	28.59 ± 0.24^c^	26.97 ± 0.98^c^	17.84 ± 0.24^c^
Ammonium sulfate	Leaves	14.62 ± 0.22^c^	35.37 ± 0.35^c^	10.78 ± 0.08^c^	40.12 ± 0.35^c^	16.39 ± 0.58^c^	29.37 ± 0.35^c^
	Stems	21.92 ± 0.33^c^	23.58 ± 0.24^c^	15.27 ± 0.12^c^	28.33 ± 0.24^c^	27.38 ± 1.00^c^	17.58 ± 0.24^c^
Ammonium nitrate	Leaves	14.31 ± 0.22^c^	36.12 ± 0.36^c^	10.59 ± 0.08^c^	40.87 ± 0.36^c^	15.98 ± 0.56^c^	30.12 ± 0.36^c^
	Stems	21.47 ± 0.33^c^	24.08 ± 0.24^c^	15.01 ± 0.12^c^	28.83 ± 0.24^c^	26.63 ± 0.97^c^	18.08 ± 0.24^c^

		Indomethacin				Zileuton	
STD		6.73 ± 0.05^b^	76.77 ± 0.47^a^	5.31 ± 0.07^b^	81.52 ± 0.47^b^	6.79 ± 0.13^b^	70.77 ± 0.47^a^

Data presented as mean ± SE, n = 3, Different superscript letters within each column indicate significant differences among treatments at *p* < 0.05.

#### Cytotoxic activity and enzymatic assays

**Fig. 2:**
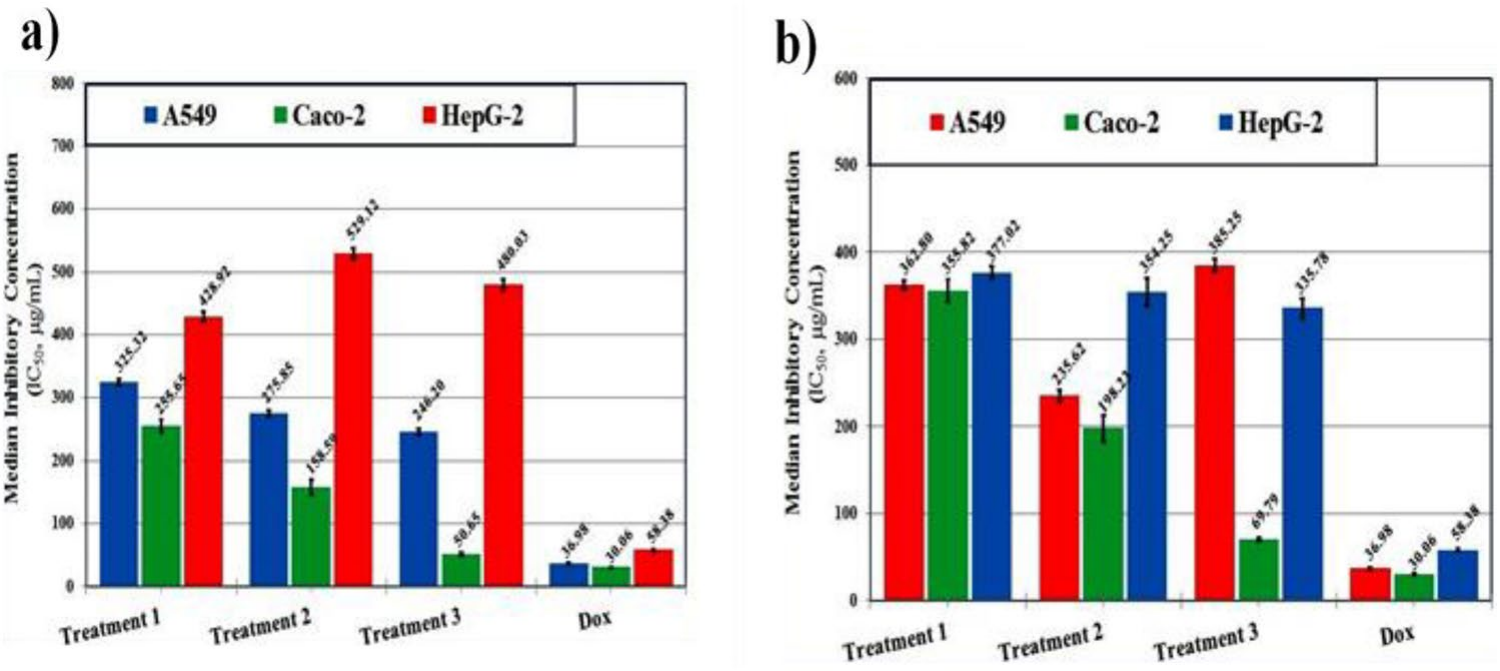
The in vitro cytotoxic activities against human hepatocellular carcinoma (HepG-2), colon (Caco-2) and lung (A549) cancer cell lines of (**a**) *C. olitorius* leaves and (**b**) stems in the three agricultural treatments and compared to Doxorubicin as standard.

The cytotoxic effects of *C. olitorius* extracts were evaluated against three human cancer cell lines (A549, Caco-2, and HepG-2). Among the different agricultural treatments, the leaf and stem extracts obtained after the third treatment demonstrated the highest cytotoxic potential, particularly against Caco-2 cells, with IC₅₀ values of 50.65 ± 2.84 and 69.79 ± 2.05 µg/mL, respectively. In contrast, extracts exhibited only weak activity against A549 and HepG-2 cells, with IC₅₀ values consistently above 200 µg/mL, indicating limited efficacy against these lines. As expected, the reference drug doxorubicin exerted potent cytotoxic activity across all tested cancer cell lines, with markedly lower IC₅₀ values (36.98 ± 0.92, 30.06 ± 0.74, and 58.38 ± 1.78 µg/mL for A549, Caco-2, and HepG-2, respectively) compared with the plant extracts (Fig. 2).

**Fig. 3:**
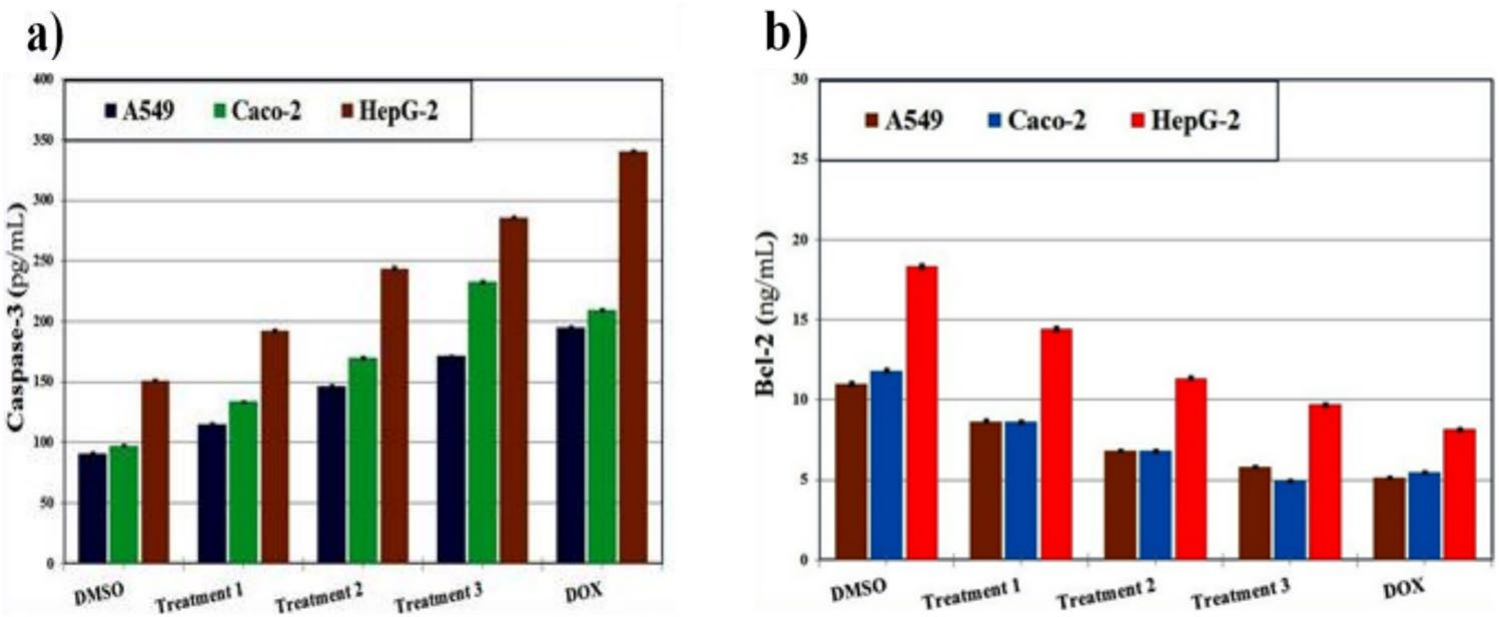
The enzymatic assay showing the effect of extracts from *C. olitorius* leaves in the three agricultural treatments on (**a**) Caspase-3 and (**b**) Bcl-2 of human hepatocellular carcinoma (HepG-2), colon (Caco-2), and lung (A549) cancer cell lines. The values were given as mean ± SE.

**Fig. 4:**
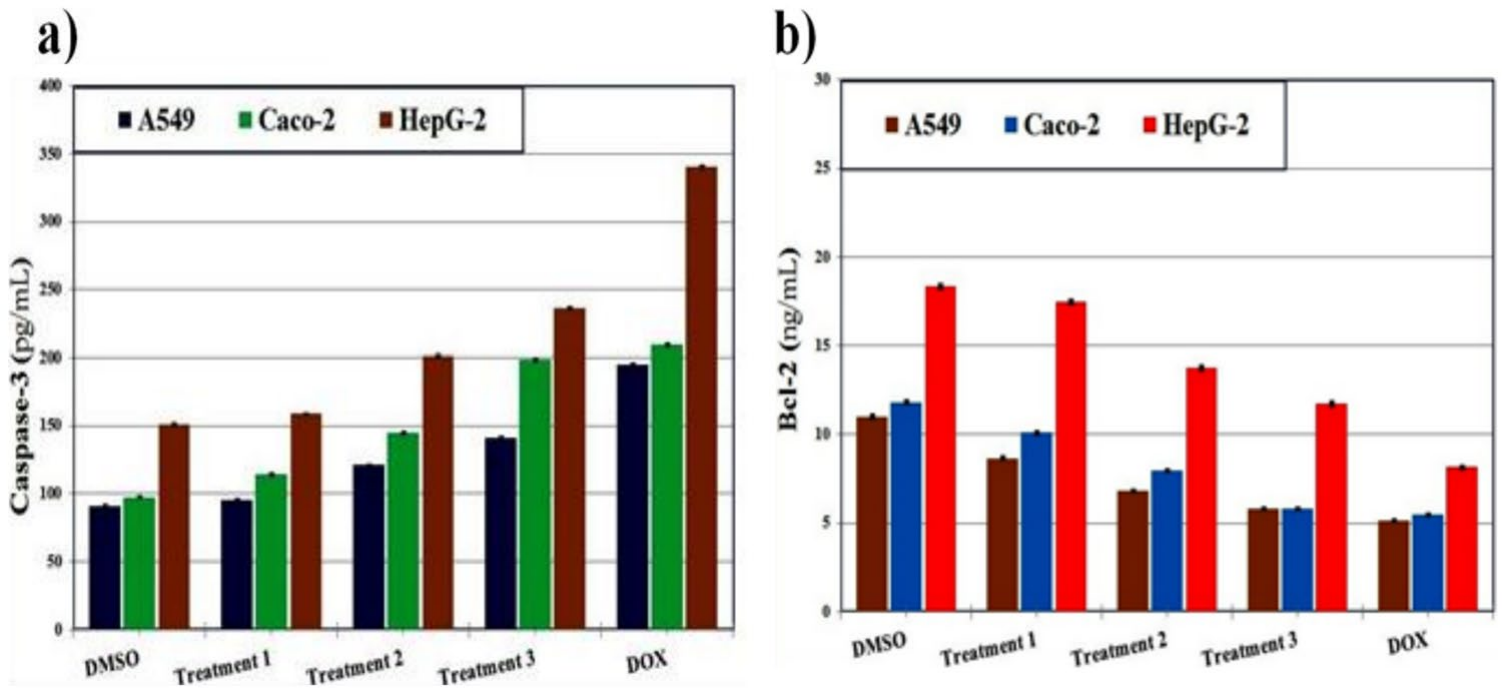
The enzymatic assay showing the effect of extracts from *C. olitorius* stems in the three agricultural treatments on (**a**) Caspase-3 and (**b**) Bcl-2 of human hepatocellular carcinoma (HepG-2), colon (Caco-2), and lung (A549) cancer cell lines. The values were given as mean ± SE.

To assess safety and selectivity, the cytotoxic activity of *C. olitorius* extracts was further examined in normal human dermal fibroblasts (HDF). All extracts displayed minimal toxicity toward normal cells, with IC₅₀ values exceeding 300 µg/mL. This contrasted sharply with their activity against cancer cells, particularly Caco-2, resulting in selectivity indices (SI) above 5. Conversely, doxorubicin exhibited strong cytotoxicity toward HDF cells, confirming its narrow therapeutic index. To further elucidate the underlying mechanisms of cytotoxicity, apoptosis-associated markers were examined. Treatment with leaf extracts from the third agricultural cycle significantly enhanced caspase-3 activity (171.34 ± 0.36, 232.66 ± 0.49, and 285.50 ± 0.60 pg/mL in A549, Caco-2, and HepG-2 cells, respectively) while concomitantly reducing Bcl-2 levels (5.83 ± 0.04, 4.96 ± 0.03, and 9.71 ± 0.07 ng/mL). A similar trend was observed with stem extracts, which increased caspase-3 activity (141.66 ± 0.30, 198.70 ± 0.42, and 236.04 ± 0.50 pg/mL) and decreased Bcl-2 expression (7.05 ± 0.05, 5.81 ± 0.04, and 11.75 ± 0.08 ng/mL) relative to untreated controls (Figs. 3 and 4). These results suggest activation of apoptotic pathways as a possible mechanism underlying the cytotoxic effects. In line with its higher potency, doxorubicin induced the strongest apoptotic response, reflected by elevated caspase-3 activity (195.21 ± 0.41, 209.85 ± 0.44, and 340.40 ± 0.72 pg/mL) and markedly reduced Bcl-2 levels (5.12 ± 0.04, 5.50 ± 0.04, and 8.15 ± 0.06 ng/mL) in A549, Caco-2, and HepG-2 cells, respectively.

Collectively, these findings indicate that *C. olitorius* extracts, especially those from the third treatment cycle, possess selective anticancer activity with a more favorable safety profile than doxorubicin, supporting their potential as promising candidates for anticancer drug development.

**Correlation analysis** (Table 11) revealed strong and statistically significant positive correlations between total phenolic and flavonoid contents and antioxidant activity (*r* = 0.91–0.93, *p* < 0.05). Similarly, significant associations were observed with anti-diabetic (*r* = 0.74–0.86, *p* < 0.05) and anti-Alzheimer’s activities (*r* = 0.65–0.82, *p* < 0.05). In contrast, correlations with anti-arthritic and anti-inflammatory activities were weak and not significant. Importantly, negative correlations were observed between phytochemical levels and IC₅₀ values for Caco-2 cytotoxicity (*r* = − 0.59 to − 0.71, *p* < 0.05), suggesting that higher levels of polyphenols and flavonoids contribute to the observed anticancer activity.

**Table 11:** Correlation coefficients (*r*) between phytochemical contents (polyphenols, tannins, flavonoids) and biological activities of *C. olitorius* extracts.

Phytochemical	Antioxidant (TAC, IRP, DPPH, ABTS)	Anti-Alzheimer (AChE inhibition)	Anti-diabetic (α-amylase, α-glucosidase)	Anti-arthritic (protein denaturation, proteinase inhibition)	Anti-inflammatory (COX-1, COX-2, 5-LOX)	Cytotoxicity (Caco-2 IC₅₀)
Polyphenols	0.91**	0.76*	0.81**	0.30 (ns)	0.42 (ns)	− 0.68*
Tannins	0.88**	0.65*	0.74*	0.25 (ns)	0.39 (ns)	− 0.59*
Flavonoids	0.93**	0.82**	0.86**	0.28 (ns)	0.44 (ns)	− 0.71**

ns = not significant; *p < 0.05.

## Discussion

The present study demonstrates that both the phytochemical composition and biological activities of *C. olitorius* are strongly influenced by the type of nitrogen source and the use of algal biostimulants. A clear trend was observed in which the leaves accumulated higher concentrations of sterols, terpenes, fatty acids, polyphenols, tannins, and flavonoids than stems, confirming that leaves are more metabolically active sites for secondary metabolite biosynthesis. Moreover, among the tested agricultural treatments, ammonium nitrate proved to be the most effective in enhancing sterols, terpenes, fatty acids, pigments, and other bioactive compounds, thereby supporting its role in promoting secondary metabolite accumulation. This finding is in line with earlier reports, such as^60^, who showed that combining nitrogen fertilizers with bio-fertilizers improved oil composition in *Nigella sativa*. The improved chemical profile under ammonium nitrate treatment can be attributed to the higher nitrogen availability, which not only supports primary metabolism but also indirectly stimulates lipid and pigment biosynthesis.

In addition to inorganic nitrogen, the role of bio-fertilizers and algal biostimulants must also be highlighted. Bio-fertilizers are known to improve nitrogen retention in the soil in the form of NH₄⁺, which facilitates nutrient uptake^61^. Similarly, they contribute to enriching soil quality and microbial diversity, thereby enhancing plant growth and metabolite accumulation^62^. Algal extracts, particularly those derived from *Chlorella vulgaris*, provide additional benefits by mitigating salinity stress^29,63^, supplying micronutrients and amino acids that stimulate growth^64,65^, and even serving as biological control agents against pathogens^66,67^. Taken together, these findings highlight the synergistic role of ammonium nitrate and algal biostimulators in improving both the growth performance and phytochemical profile of *C. olitorius*.

The effect of these agricultural practices was also evident in pigment accumulation. The increase in chlorophyll and carotenoid contents under ammonium nitrate treatment demonstrates the positive influence of this fertilizer on pigment biosynthesis. Interestingly, these values were higher than those reported for wild *C. olitorius* by Helaly et al.^68^, a difference that may be explained by variations in cultivation practices and environmental conditions^69^. Similar improvements in chlorophyll levels following bio-fertilization have also been observed in *Anethum graveolens*^70^ and *Cuminum cyminum*^60^. Moreover, Youssef et al.^71^ reported synergistic effects of organic and bio-fertilizers on chlorophyll accumulation, further supporting the present results. Carotenoid concentrations matched the range reported by Hosen et al.^72^, indicating that fertilization strategies play a decisive role in determining pigment levels. Since chlorophylls and carotenoids contribute not only to photosynthetic efficiency but also to nutritional and health-promoting properties, optimizing agricultural practices and raising awareness of postharvest handling are essential to preserve pigment quality.

The same pattern was reflected in the accumulation of polyphenols and related bioactive compounds. Leaves contained higher concentrations of phenolics, tannins, and flavonoids than stems, which agrees with the findings of Biswas et al.^73^, who also reported greater metabolite abundance in aerial parts. Once again, ammonium nitrate appeared to be the most effective treatment, confirming previous studies that highlighted the benefits of combining inorganic nitrogen with bio-fertilizers to enhance plant growth and metabolite production^74^. Seasonal and environmental influences may also contribute to variation in phenolic levels, as indicated by Yakoub et al.^75^. The abundance of phenolic acids, such as gallic and chlorogenic acids, and flavonoids, including quercetin, kaempferol, and naringenin, particularly in nitrate-treated plants, underscores the nutritional and functional significance of *C. olitorius* as a leafy vegetable.

The improvement in phytochemical content was mirrored by strong antioxidant and radical scavenging activities. The highest activities, including total antioxidant capacity, iron-reducing power, and radical scavenging assays, were recorded in leaves treated with ammonium nitrate. This direct correlation between chemical composition and bioactivity supports the established role of phenolics and flavonoids in antioxidant defense^76^. Consistent with this observation, Ouattar et al.^77^ reported that phenolic acids and flavonoids can efficiently neutralize reactive oxygen and nitrogen species. Although ascorbic acid exhibited the strongest activity overall, the extracts of *C. olitorius* still demonstrated remarkable antioxidant capacity, further reinforcing their potential as natural sources of health-promoting antioxidants.

The relationship between phytochemical richness and bioactivity was also evident in the neuroprotective assays. Extracts from ammonium nitrate-treated leaves demonstrated promising anti-Alzheimer’s potential, as shown by their ability to inhibit acetylcholinesterase (AChE). This activity paralleled their antioxidant performance, suggesting that reducing oxidative stress may contribute to **neuroprotection**^78^. The activity can be partly attributed to phenolic compounds, as Afzal et al.^79^ reported that phenolics interact with amino acid residues of the enzyme via hydrogen bonding and hydrophobic interactions, a finding further supported by Younis et al.^80^, who demonstrated the antioxidant, antidiabetic, and anticholinesterase effects of *Cynara cardunculus* leaves. Atalar et al.^81^ further emphasized that multiple hydroxyl groups in phenolics increase binding affinity and inhibitory potential. Collectively, these observations suggest that *C. olitorius*, particularly when cultivated with ammonium nitrate, may serve as a valuable complementary source of natural anti-Alzheimer’s agents alongside conventional therapies such as donepezil.

In addition to neuroprotective activity, the extracts exhibited significant antidiabetic effects through inhibition of α-amylase and α-glucosidase. Leaf extracts again showed stronger effects than stems, reflecting their higher metabolite concentrations. These results are consistent with those of Oboh et al.^82^ and Ghellam et al.^83^, who reported strong antidiabetic activity for *C. olitorius* due to its phenolic content. Aboulthana et al.^84^ also highlighted the role of tannins and phenolic acids in reducing postprandial hyperglycemia by inhibiting carbohydrate-hydrolyzing enzymes. According to Abdelazeem et al.^85^, the inhibition mechanism largely depends on the number and arrangement of hydroxyl groups, which enhance binding to enzyme active sites. Similarly, El Sawi et al.^86^ demonstrated that dihydroxyl substitution at the C-3′ and C-4′ positions of the flavonoid system plays a critical role in α-glucosidase inhibition by modulating the electron cloud distribution on the B ring, thereby facilitating hydrogen bond formation with the enzyme’s binding site. In addition, isoflavones have shown notable antidiabetic properties, particularly through promoting β-cell proliferation and regulating insulin secretion. Although acarbose remained the most potent inhibitor, extracts of *C. olitorius* exhibited comparable activity, highlighting their potential as natural, plant-derived alternatives or complementary agents in diabetes management.

However, when tested for anti-arthritic and anti-inflammatory potential, the extracts did not show significant activity under the studied conditions. Neither protein denaturation nor proteinase inhibition was affected, unlike diclofenac sodium, which strongly inhibited both pathways^87^. Similarly, weak inhibition of COX-1, COX-2, and 5-LOX enzymes suggested limited effects on the arachidonic acid cascade, in contrast to indomethacin and zileuton, which acted as strong inhibitors^88,89^. These findings indicate that the anti-inflammatory potential of *C. olitorius* may not rely on direct enzyme inhibition but instead on alternative mechanisms, such as antioxidant-mediated suppression of inflammatory signaling or modulation of cytokine expression.

Finally, the extracts showed selective cytotoxicity, particularly against Caco-2 cells, with the strongest effect observed in ammonium nitrate-treated leaves and stems. This activity may be explained by their higher phenolic and flavonoid content, which are known to interfere with cell cycle regulation and suppress tumor cell proliferation^90–92^. Moreover, the incorporation of cytotoxicity testing on normal human dermal fibroblasts (HDF) added an essential safety dimension to the study, demonstrating that the extracts exhibited minimal toxicity toward normal cells, thereby reinforcing their selectivity and therapeutic relevance. The induction of apoptosis was further supported by the increase in caspase-3 and decrease in Bcl-2 levels, which is consistent with the mechanism proposed by Shaikh et al.^93^. Although doxorubicin exhibited the most pronounced cytotoxic effect^94^, the extracts contain flavonoids, are capable of upregulating caspase-3 and downregulating Bcl-2 and Bax^95^. This suggests that, while less potent than conventional chemotherapy drugs, *C. olitorius* may nevertheless represent a valuable complementary source of bioactive compounds with selective efficacy against colon cancer cells. Although the results are promising, this study is limited by its in vitro design, and further in vivo and clinical investigations are required.

## Conclusion

The combined application of biofertilizer and ammonium nitrate significantly enhanced the accumulation of essential phytoconstituents—including polyphenols, tannins, and flavonoids—more effectively than other treatments. Both leaves and stems contained diverse bioactive compounds such as sterols, terpenes, fatty acids, chlorophylls, carotenoids, phenolics, tannins, and flavonoids. Extracts from plants subjected to the third treatment exhibited the strongest antioxidant, neuroprotective, antidiabetic, and cytotoxic activities. Conversely, anti-arthritic and anti-inflammatory activities were not markedly affected. Overall, these findings highlight *C. olitorius* as a rich source of phytochemicals with promising biological activities, particularly when cultivated under optimized fertilization strategies. Further research is warranted to identify specific active compounds and clarify their mechanisms of action.

## Supplementary Information

Supplementary Information.

## Data Availability

All data collected during this investigation are incorporated in the publication.
