# Supplementary material for: The influence of algal biostimulator and nitrogen source on the phytochemical composition and biological properties of Corchorus olitorius leaves and stems
Source: Sci Rep. 2026 Mar 12;16:8948. doi: 10.1038/s41598-026-39774-z (PMC12988189; doi:10.1038/s41598-026-39774-z)
Supplement: Supplementary file 1 — Supplementary Information. [file 41598_2026_39774_MOESM1_ESM.doc]

**The influence of algal Biostimulator and nitrogen source on the phytochemical composition and biological properties of *Corchorus olitorius* leaves and stems**

**Wael Mahmoud Aboulthana^1^, Amal M. El-Feky^2*^, Nagwa I. Omar^1^, Abo El-Khair B. El-Sayed^3^**

^1^Biochemistry Department, Biotechnology Research Institute, National Research Centre, 33 El Bohouth St., P.O. 12622, Dokki, Giza, Egypt.

^2^Pharmacognosy Department, Pharmaceutical and Drug Industries Research Institute, National Research Centre, 33 El Bohouth St., P.O. 12622, Dokki, Giza, Egypt.

^3^Algal Biotechnology Unit, Department of Fertilization Technology, Biological, and Agricultural Research Institution, National Research Centre, 33 El Bohouth St., P.O. 12622, Dokki, Giza, Egypt.

**For Correspondence:* **Amal M. El-Feky,**[*ammelfeky@hotmail.com*](mailto:ammelfeky@hotmail.com)

**Supplementary Table 1:** The *in vitro* cytotoxic activities of extracts from *C. olitorius* leaves and stems in the three agricultural treatments against human hepatocellular carcinoma (HepG-2), colon (Caco-2) and lung (A549) cancer cell lines.

|  | | **Median Inhibitory Concentration (**IC_50_**)**  **(**µg/mL**)** | | | |
| --- | --- | --- | --- | --- | --- |
|  |  | **A549** | **Caco-2** | **HepG-2** | **HDF (Normal cells)** |
| **First Treatment** | **Leaves** | 325.32 ± 3.70 | 255.65 ± 10.60 | 428.92 ± 7.03 | >300 ± NA |
|  | **Stem** | 362.80 ± 5.32 | 355.82 ± 12.64 | 377.02 ± 6.75 | >300 ± NA |
| **Second Treatment** | **Leaves** | 275.85 ± 4.59 | 158.59 ± 12.04 | 529.12 ± 8.73 | >300 ± NA |
|  | **Stem** | 235.62 ± 5.94 | 198.23 ± 15.05 | 354.25 ± 15.41 | >300 ± NA |
| **Third Treatment** | **Leaves** | 246.20 ± 5.32 | **50.65 ± 2.84** | 480.03 ± 7.86 | >300 ± NA |
|  | **Stem** | 385.25 ± 6.54 | **69.79 ± 2.05** | 335.78 ± 11.58 | >300 ± NA |
| **STD**  **(Doxorubicin)** | | **36.98 ± 0.92** | **30.06 ± 0.74** | **58.38 ± 1.78** | 42.15 ± 1.05 |

The data was calculated from n=3/extract and shown as mean ± SE.

NA = not applicable for SE, as values exceeded the assay limit.

**Supplementary Table 2:** The *in vitro* cytotoxic activity of *C. olitorius* leaves and stems in the three agricultural treatments against human lung cancer (A549) cell line and compared to Doxorubicin as standard.

| **First Treatment** | | | | | | | | | | | |
| --- | --- | --- | --- | --- | --- | --- | --- | --- | --- | --- | --- |
| **Leaves** | | | | | | | | | | | |
| **Conc. (**μg/mL**)** | **0.00** | **31.13** | | **62.50** | | **125.00** | | **250.00** | | **500.00** | |
| Mean OD | 0.36 | 0.28 | | 0.23 | | 0.16 | | 0.10 | | 0.08 | |
| Viability % | 100.00 | 95.02 | | 87.61 | | 74.33 | | 45.13 | | 34.49 | |
| Cytotoxicity % | 0.00 | 4.98 | | 12.39 | | 25.67 | | 54.87 | | 65.51 | |
| **IC_50_ (**μg/mL**)** | **325.32 ± 3.70** | | | | | | | | | | |
| **Stem** | | | | | | | | | | | |
| **Conc. (**μg/mL**)** | **0.00** | | **31.13** | | **62.50** | | **125.00** | | **250.00** | | **500.00** |
| Mean OD | 0.34 | | 0.29 | | 0.26 | | 0.18 | | 0.11 | | 0.07 |
| Viability % | 100.00 | | 91.13 | | 79.91 | | 67.53 | | 48.51 | | 29.94 |
| Cytotoxicity % | 0.00 | | 8.87 | | 20.09 | | 32.47 | | 51.49 | | 70.06 |
| **IC_50_ (**μg/mL**)** | **362.80 ± 5.32** | | | | | | | | | | |

| **Second Treatment** | | | | | | | | | | | |
| --- | --- | --- | --- | --- | --- | --- | --- | --- | --- | --- | --- |
| **Leaves** | | | | | | | | | | | |
| **Conc. (**μg/mL**)** | **0.00** | **31.13** | | **62.50** | | **125.00** | | **250.00** | | **500.00** | |
| Mean OD | 0.31 | 0.26 | | 0.23 | | 0.15 | | 0.08 | | 0.05 | |
| Viability % | 100.00 | 87.85 | | 78.99 | | 68.75 | | 55.75 | | 48.51 | |
| Cytotoxicity % | 0.00 | 12.15 | | 21.01 | | 31.25 | | 44.25 | | 51.49 | |
| **IC_50_ (**μg/mL**)** | **275.85 ± 4.59** | | | | | | | | | | |
| **Stem** | | | | | | | | | | | |
| **Conc. (**μg/mL**)** | **0.00** | | **31.13** | | **62.50** | | **125.00** | | **250.00** | | **500.00** |
| Mean OD | 0.35 | | 0.29 | | 0.24 | | 0.15 | | 0.09 | | 0.08 |
| Viability % | 100.00 | | 85.85 | | 75.29 | | 61.75 | | 52.75 | | 44.51 |
| Cytotoxicity % | 0.00 | | 14.15 | | 24.71 | | 38.25 | | 47.25 | | 55.49 |
| **IC_50_ (**μg/mL**)** | **235.62 ± 5.94** | | | | | | | | | | |

| **Third Treatment** | | | | | | | | | | | |
| --- | --- | --- | --- | --- | --- | --- | --- | --- | --- | --- | --- |
| **Leaves** | | | | | | | | | | | |
| **Conc. (**μg/mL**)** | **0.00** | **31.13** | | **62.50** | | **125.00** | | **250.00** | | **500.00** | |
| Mean OD | 0.33 | 0.28 | | 0.25 | | 0.17 | | 0.10 | | 0.06 | |
| Viability % | 100.00 | 81.78 | | 71.75 | | 62.85 | | 54.75 | | 43.71 | |
| Cytotoxicity % | 0.00 | 18.22 | | 28.25 | | 37.15 | | 45.25 | | 56.29 | |
| **IC_50_ (**μg/mL**)** | **246.20 ± 5.32** | | | | | | | | | | |
| **Stem** | | | | | | | | | | | |
| **Conc. (**μg/mL**)** | **0.00** | | **31.13** | | **62.50** | | **125.00** | | **250.00** | | **500.00** |
| Mean OD | 0.40 | | 0.34 | | 0.29 | | 0.20 | | 0.14 | | 0.02 |
| Viability % | 100.00 | | 91.75 | | 82.55 | | 71.95 | | 65.77 | | 48.25 |
| Cytotoxicity % | 0.00 | | 8.25 | | 17.45 | | 28.05 | | 34.23 | | 51.75 |
| **IC_50_ (**μg/mL**)** | **385.25 ± 6.54** | | | | | | | | | | |

| **DOX** | | | | | | |
| --- | --- | --- | --- | --- | --- | --- |
| **Conc. (**μg/mL**)** | **0.00** | **6.25** | **12.50** | **25.00** | **50.00** | **100.00** |
| Mean OD | 0.26 | 0.21 | 0.16 | 0.12 | 0.09 | 0.75 |
| Viability % | 100.00 | 87.67 | 76.05 | 65.22 | 35.58 | 18.72 |
| Cytotoxicity % | 0.00 | 12.33 | 23.95 | 34.78 | 64.42 | 81.28 |
| **IC_50_ (**μg/mL**)** | **36.98 ± 0.92** | | | | | |

**Supplementary Table 3:** The *in vitro* cytotoxic activity of *C. olitorius* leaves and stems in the three agricultural treatments against human colon cancer (Caco2) cell line and compared to Doxorubicin as standard.

| **First Treatment** | | | | | | | | | | | |
| --- | --- | --- | --- | --- | --- | --- | --- | --- | --- | --- | --- |
| **Leaves** | | | | | | | | | | | |
| **Conc. (**μg/mL**)** | **0.00** | **6.25** | | **12.50** | | **25.00** | | **50.00** | | **100.00** | |
| Mean OD | 0.39 | 0.26 | | 0.20 | | 0.10 | | 0.07 | | 0.04 | |
| Viability % | 100.00 | 65.72 | | 52.25 | | 43.25 | | 40.97 | | 31.25 | |
| Cytotoxicity % | 0.00 | 34.28 | | 47.75 | | 56.75 | | 59.03 | | 68.75 | |
| **IC_50_ (**μg/mL**)** | **255.65 ± 10.60** | | | | | | | | | | |
| **Stem** | | | | | | | | | | | |
| **Conc. (**μg/mL**)** | **0.00** | | **6.25** | | **12.50** | | **25.00** | | **50.00** | | **100.00** |
| Mean OD | 0.30 | | 0.23 | | 0.18 | | 0.11 | | 0.08 | | 0.04 |
| Viability % | 100.00 | | 81.72 | | 58.25 | | 42.25 | | 37.45 | | 28.77 |
| Cytotoxicity % | 0.00 | | 18.28 | | 41.75 | | 57.75 | | 62.55 | | 71.23 |
| **IC_50_ (**μg/mL**)** | **355.82 ± 12.64** | | | | | | | | | | |

| **Second Treatment** | | | | | | | | | | | |
| --- | --- | --- | --- | --- | --- | --- | --- | --- | --- | --- | --- |
| **Leaves** | | | | | | | | | | | |
| **Conc. (**μg/mL**)** | **0.00** | **6.25** | | **12.50** | | **25.00** | | **50.00** | | **100.00** | |
| Mean OD | 0.40 | 0.28 | | 0.21 | | 0.12 | | 0.09 | | 0.04 | |
| Viability % | 100.00 | 68.25 | | 51.55 | | 45.78 | | 36.25 | | 27.95 | |
| Cytotoxicity % | 0.00 | 31.75 | | 48.45 | | 54.22 | | 63.75 | | 72.05 | |
| **IC_50_ (**μg/mL**)** | **158.59 ± 12.04** | | | | | | | | | | |
| **Stem** | | | | | | | | | | | |
| **Conc. (**μg/mL**)** | **0.00** | | **6.25** | | **12.50** | | **25.00** | | **50.00** | | **100.00** |
| Mean OD | 0.29 | | 0.22 | | 0.17 | | 0.10 | | 0.07 | | 0.03 |
| Viability % | 100.00 | | 78.25 | | 62.75 | | 50.17 | | 35.85 | | 29.25 |
| Cytotoxicity % | 0.00 | | 21.75 | | 37.25 | | 49.83 | | 64.15 | | 70.75 |
| **IC_50_ (**μg/mL**)** | **198.23 ± 15.05** | | | | | | | | | | |

| **Third Treatment** | | | | | | | | | | | |
| --- | --- | --- | --- | --- | --- | --- | --- | --- | --- | --- | --- |
| **Leaves** | | | | | | | | | | | |
| **Conc. (**μg/mL**)** | **0.00** | **6.25** | | **12.50** | | **25.00** | | **50.00** | | **100.00** | |
| Mean OD | 0.33 | 0.24 | | 0.20 | | 0.12 | | 0.08 | | 0.04 | |
| Viability % | 100.00 | 68.04 | | 54.57 | | 43.63 | | 31.17 | | 25.43 | |
| Cytotoxicity % | 0.00 | 31.96 | | 45.43 | | 56.37 | | 68.83 | | 74.57 | |
| **IC_50_ (**μg/mL**)** | **50.65 ± 2.84** | | | | | | | | | | |
| **Stem** | | | | | | | | | | | |
| **Conc. (**μg/mL**)** | **0.00** | | **6.25** | | **12.50** | | **25.00** | | **50.00** | | **100.00** |
| Mean OD | 0.29 | | 0.22 | | 0.17 | | 0.10 | | 0.06 | | 0.01 |
| Viability % | 100.00 | | 89.99 | | 72.16 | | 57.7 | | 41.23 | | 33.64 |
| Cytotoxicity % | 0.00 | | 10.01 | | 27.84 | | 42.30 | | 58.77 | | 66.36 |
| **IC_50_ (**μg/mL**)** | **69.79 ± 2.05** | | | | | | | | | | |

| **DOX** | | | | | | |
| --- | --- | --- | --- | --- | --- | --- |
| **Conc. (**μg/mL**)** | **0.00** | **6.25** | **12.50** | **25.00** | **50.00** | **100.00** |
| Mean OD | 0.37 | 0.29 | 0.22 | 0.14 | 0.08 | 0.03 |
| Viability % | 100.00 | 86.28 | 67.27 | 45.37 | 29.64 | 17.69 |
| Cytotoxicity % | 0.00 | 13.72 | 32.73 | 54.63 | 70.36 | 82.31 |
| **IC_50_ (**μg/mL**)** | **30.06 ± 0.74** | | | | | |

**Supplementary Table 4:** The *in vitro* cytotoxic activity of *C. olitorius* leaves and stems in the three agricultural treatments against human liver carcinoma (HepG2) cell line and compared to Doxorubicin as standard.

| **First Treatment** | | | | | | | | | | | |
| --- | --- | --- | --- | --- | --- | --- | --- | --- | --- | --- | --- |
| **Leaves** | | | | | | | | | | | |
| **Conc. (**μg/mL**)** | **0.00** | **31.13** | | **62.50** | | **125.00** | | **250.00** | | **500.00** | |
| Mean OD | 0.41 | 0.39 | | 0.36 | | 0.27 | | 0.14 | | 0.06 | |
| Viability % | 100.00 | 94.49 | | 75.77 | | 60.58 | | 43.29 | | 35.32 | |
| Cytotoxicity % | 0.00 | 5.51 | | 24.23 | | 39.42 | | 56.71 | | 64.68 | |
| **IC_50_ (**μg/mL**)** | **428.92 ± 7.03** | | | | | | | | | | |
| **Stem** | | | | | | | | | | | |
| **Conc. (**μg/mL**)** | **0.00** | | **31.13** | | **62.50** | | **125.00** | | **250.00** | | **500.00** |
| Mean OD | 0.37 | | 0.27 | | 0.21 | | 0.14 | | 0.09 | | 0.04 |
| Viability % | 99.21 | | 79.56 | | 63.61 | | 45.45 | | 37.09 | | 30.71 |
| Cytotoxicity % | 0.79 | | 20.44 | | 36.39 | | 54.55 | | 62.91 | | 69.29 |
| **IC_50_ (**μg/mL**)** | **377.02 ± 6.75** | | | | | | | | | | |

| **Second Treatment** | | | | | | | | | | | |
| --- | --- | --- | --- | --- | --- | --- | --- | --- | --- | --- | --- |
| **Leaves** | | | | | | | | | | | |
| **Conc. (**μg/mL**)** | **0.00** | **31.13** | | **62.50** | | **125.00** | | **250.00** | | **500.00** | |
| Mean OD | 0.42 | 0.31 | | 0.22 | | 0.16 | | 0.10 | | 0.05 | |
| Viability % | 100.00 | 83.54 | | 66.79 | | 47.73 | | 38.94 | | 32.24 | |
| Cytotoxicity % | 0.00 | 16.46 | | 33.21 | | 52.27 | | 61.06 | | 67.76 | |
| **IC_50_ (**μg/mL**)** | **529.12 ± 8.73** | | | | | | | | | | |
| **Stem** | | | | | | | | | | | |
| **Conc. (**μg/mL**)** | **0.00** | | **31.13** | | **62.50** | | **125.00** | | **250.00** | | **500.00** |
| Mean OD | 0.39 | | 0.29 | | 0.20 | | 0.15 | | 0.09 | | 0.05 |
| Viability % | 100.00 | | 87.71 | | 70.13 | | 50.11 | | 40.89 | | 33.86 |
| Cytotoxicity % | 0.00 | | 12.29 | | 29.87 | | 49.89 | | 59.11 | | 66.14 |
| **IC_50_ (**μg/mL**)** | **354.25 ± 15.41** | | | | | | | | | | |

| **Third Treatment** | | | | | | | | | | | |
| --- | --- | --- | --- | --- | --- | --- | --- | --- | --- | --- | --- |
| **Leaves** | | | | | | | | | | | |
| **Conc. (**μg/mL**)** | **0.00** | **31.13** | | **62.50** | | **125.00** | | **250.00** | | **500.00** | |
| Mean OD | 0.38 | 0.29 | | 0.20 | | 0.15 | | 0.09 | | 0.05 | |
| Viability % | 100.00 | 76.27 | | 60.98 | | 43.58 | | 35.55 | | 29.44 | |
| Cytotoxicity % | 0.00 | 23.73 | | 39.02 | | 56.42 | | 64.45 | | 70.56 | |
| **IC_50_ (**μg/mL**)** | **480.03 ± 7.86** | | | | | | | | | | |
| **Stem** | | | | | | | | | | | |
| **Conc. (**μg/mL**)** | **0.00** | | **31.13** | | **62.50** | | **125.00** | | **250.00** | | **500.00** |
| Mean OD | 0.41 | | 0.38 | | 0.36 | | 0.26 | | 0.14 | | 0.06 |
| Viability % | 100.00 | | 93.02 | | 88.14 | | 78.84 | | 66.05 | | 45.86 |
| Cytotoxicity % | 0.00 | | 6.98 | | 11.86 | | 21.16 | | 33.95 | | 54.14 |
| **IC_50_ (**μg/mL**)** | **335.78 ± 11.58** | | | | | | | | | | |

| **DOX** | | | | | | |
| --- | --- | --- | --- | --- | --- | --- |
| **Conc. (**μg/mL**)** | **0.00** | **6.25** | **12.50** | **25.00** | **50.00** | **100.00** |
| Mean OD | 0.33 | 0.24 | 0.21 | 0.12 | 0.08 | 0.05 |
| Viability % | 100.00 | 65.27 | 55.92 | 39.8 | 29.53 | 15.97 |
| Cytotoxicity % | 0.00 | 34.73 | 44.08 | 60.20 | 70.47 | 84.03 |
| **IC_50_ (**μg/mL**)** | **58.38 ± 1.78** | | | | | |

**Supplementary Table 5:** The enzymatic assay values after the treatment of human liver carcinoma (HepG2), colon cancer (Caco-2), and lung cancer (A549) cell lines with extracts from *C. olitorius* leaves and stems in the three agricultural treatments at the IC_50_ value and compared to Doxorubicin used as standard.

|  | | **A549** | | **Caco-2** | | **HepG-2** | |
| --- | --- | --- | --- | --- | --- | --- | --- |
|  |  | **Caspase-3 (**pg/mL**)** | **Bcl-2**  **(**ng/mL**)** | **Caspase-3 (**pg/mL**)** | **Bcl-2**  **(**ng/mL**)** | **Caspase-3 (**pg/mL**)** | **Bcl-2**  **(**ng/mL**)** |
| **DMSO** | | 90.80 ± 0.19 | 11.00 ± 0.08 | 97.61 ± 0.21 | 11.82 ± 0.08 | 151.29 ± 0.32 | 18.33 ± 0.13 |
| **Treatment 1** | **Leaves** | 115.31 ± 0.24 | 8.66 ± 0.06 | 133.72 ± 0.28 | 8.63 ± 0.06 | 192.14 ± 0.40 | 14.43 ± 0.10 |
|  | **Stems** | 95.34 ± 0.20 | 10.48 ± 0.07 | 114.20 ± 0.24 | 10.11 ± 0.07 | 158.85 ± 0.33 | 17.45 ± 0.12 |
| **Treatment 2** | **Leaves** | 146.45 ± 0.31 | 6.82 ± 0.05 | 169.83 ± 0.36 | 6.80 ± 0.05 | 244.02 ± 0.51 | 11.36 ± 0.08 |
|  | **Stems** | 121.08 ± 0.25 | 8.25 ± 0.06 | 145.03 ± 0.30 | 7.96 ± 0.05 | 201.75 ± 0.42 | 13.74 ± 0.09 |
| **Treatment 3** | **Leaves** | 171.34 ± 0.36 | 5.83 ± 0.04 | 232.66 ± 0.49 | 4.96 ± 0.03 | 285.50 ± 0.60 | 9.71 ± 0.07 |
|  | **Stems** | 141.66 ± 0.30 | 7.05 ± 0.05 | 198.70 ± 0.42 | 5.81 ± 0.04 | 236.04 ± 0.50 | 11.75 ± 0.08 |
| **DOX** | | **195.21 ± 0.41** | **5.12 ± 0.04** | **209.85 ± 0.44** | **5.50 ± 0.04** | **340.40 ± 0.72** | **8.15 ± 0.06** |

The values were calculated from n=3/sample and given as mean ± SE.
